# Reflectance Variation within the In-Chlorophyll Centre Waveband for Robust Retrieval of Leaf Chlorophyll Content

**DOI:** 10.1371/journal.pone.0110812

**Published:** 2014-11-03

**Authors:** Jing Zhang, Wenjiang Huang, Qifa Zhou

**Affiliations:** 1 College of Life Sciences, Zhejiang University, Hangzhou, China; 2 Key Laboratory of Digital Earth Science, Institute of Remote Sensing and Digital Earth, Chinese Academy of Sciences, Beijing, China; Beijing Forestry University, China

## Abstract

The in-chlorophyll centre waveband (ICCW) (640–680 nm) is the specific chlorophyll (Chl) absorption band, but the reflectance in this band has not been used as an optimal index for non-destructive determination of plant Chl content in recent decades. This study develops a new spectral index based solely on the ICCW for robust retrieval of leaf Chl content for the first time. A glasshouse experiment for solution-culture of one chlorophyll-deficient rice mutant and six wild types of rice genotypes was conducted, and the leaf reflectance (400–900 nm) was measured with a high spectral resolution (1 nm) spectrophotometer and the contents of chlorophyll a (Chl*a*), chlorophyll b (Chl*b*) and chlorophyll a+b (Chl*t*) of the rice leaves were determined. It was found that the reflectance curves from 640 nm to 674 nm and from 675 nm to 680 nm of the low-chlorophyll mutant leaf were drastically steeper than that of the wild types in the ICCW. The new index based on the reflectance variation within ICCW, the difference of the first derivative sum within the ICCW (DFDS_ICCW), was highly sensitive (r = −0.77, n = 93, *P*<0.01) to Chl*t* while the mean reflectance (R_ICCW) in the ICCW became insensitive (*r* = −0.12, n = 93, *P*>0.05) to Chl*t* when the leaf Chl*t* was higher than 200 mg/m^2^. The best equations of R-ICCW and DFDS_ICCW yielded an RMSE of 78.7, 32.9 and 107.3 mg/m^2^, and an RMSE of 37.4, 16.0 and 45.3 mg/m^−2^, respectively, for predicting Chl*a*, Chl*b* and Chl*t*. The new index could rank in the top 10 for prediction of Chl*a* and Chl*t* as compared with the 55 existing indices. Additionally, most of the 55 existing Chl-related VIs performed robustly or strongly in simultaneous prediction of leaf Chl*a*, Chl*b* and Chl*t*.

## Introduction

Chlorophyll (Chl) a and Chl b are major constituents of the photosynthetic apparatus in higher plants. Chl a and Chl b are interconverted in the chlorophyll cycle [1]. Leaf Chl a concentration (Chl*a*) and Chl b concentration (Chl*b*) indicate a plant's photosynthetic capacity and health status, and determination of Chl*a*, Chl*b* and ratios of Chl*a* to Chl*b* are also helpful for understanding the light acclimation mechanisms in higher plants [2]. Conventionally, leaf Chl*a* and Chl*b* are determined with a traditional wet extraction analysis based on measuring the extinction of the extract at the major red absorption maxima of Chl a (∼664 nm) and b (∼647 nm) in the in-chlorophyll centre waveband (640–680 nm), and by inserting these values into simultaneous equations [2], [3]. In recent decades, there has been an increasing interest in non-destructively determining leaf and canopy Chl content by measuring leaf and canopy spectral reflectance. Particular efforts have been devoted to the development of robust algorithms for Chl*t* determination from the leaf to canopy scale [4]–[10]. Contrastingly, studies conducted for determination of individual Chl*a* or individual Chl*b* with spectral vegetation indices (VIs) are much less frequent [4], [6], [11]. Reflectance in the ICCW had been used for a long time as an indicator of chlorophyll content of leaves, but has not been used as an optimal index since Thomas and Gausman (1977) [12] found that reflectance near 675 nm became saturated at medium to high chlorophyll concentrations [6]. In recent decades, many studies have found that reflectance in the green and red-edge spectral regions was optimal for non-destructive estimation of leaf Chl content in a wide range of its variation [13]–[16]. The results of Féret et al. (2011) [17] showed that the reflectance in the red-edge and near infrared spectral regions simulated with the Prospect 5 radiative transfer model provided an accurate estimation of leaf Chl content. Recently, Main et al. (2011) [11] assessed the performance of 73 published VIs for leaf Chl estimation and also found that the indices using off-chlorophyll absorption centre wavebands (OCCW) performed better than those using ICCW. To our best knowledge, no VIs based solely on ICCW for Chl estimation have been developed since Thomas and Gausman (1977) [12] found the saturated reflection of plant leaves. Plant leaves have a reflectance minima around 675 nm, and there are substantial differences in reflectance among different wavelengths in the ICCW. Is the reflectance difference within the ICCW associated with the Chl content? This study has two objectives. The first is to examine the robustness of simultaneous estimation of Chl*a*, Chl*b* and Chl*t* with the existing Chl-related VIs and commercial chlorophyll meter readings by using a dataset of measured reflectance, Chl*a*, Chl*b* and Chl*t* of rice leaves of different genotypes including low-chlorophyll mutants (low in Chl content) at different stages. Second, we test if the reflectance difference within the ICCW is associated with the Chl content by using the constructed dataset and then solely using ICCW to develop a new VI simultaneously sensitive to Chl*a*, Chl*b* and Chl*t*.

## Materials and Methods

### 2.1. Plant materials and growth conditions

A pot experiment was conducted in a greenhouse with natural light (mean daily photosynthetically active radiation 130 µmol m^−2^ s^−1^ during the whole growth period) and controlled temperature (daily maximum 27.6°C, daily minimum 16.2°C during the rice growing period) and humidity (24.5–85.1% average daily relative humidity, RH, throughout the whole rice growing period) at Zhejiang University Experimental Farm, Hangzhou, China (30°14′ N, 120°10′ E). Six wild types of rice genotypes (IG1, IG23, IG24, DJ, NIP and ZH11) and one chlorophyll-deficient mutant (IG20) were solution-cultured according to the IRRI prescription [18], but the nitrogen level was designed as 1/5×40 mg l^−1^ (low N) and 40 mg l^−1^ (normal N), respectively, for two nitrogen treatments. The mutant ‘IG 20’ is an isogenic line of the recurrent parent “Zhefu 802” bred by China National Rice Research Institute. A completely random design with four replications was used. Each pot contained a 6.0-L nutrient solution and three seedlings. The nutrient solution was replaced as the electric conductivity decreased to half of the original. The plants were transplanted on October 1, 2013.

### 2.2. Chlorophyll meter and spectral measurements

The second uppermost leaves of each treatment were measured in situ with a SPAD 502 model chlorophyll meter (Konica Minota Inc., Japan) around the midpoint at tillering, booting and heading. After the measurement of the chlorophyll meter, the leaves were immediately sampled and stored in an ice box, and transported to the lab for leaf reflectance measurements. The reflectance of the single leaf was measured with an integrating sphere (model LISR-3100, Shimadzu Scientific Instruments Inc., Japan) coupled to a UV-3600 UV-VIS-NIR spectrophotometer (Shimadzu Scientific Instruments Inc., Japan) in the wavelength range of 400–900 nm around the midpoint of each leaf. The spectral meter has a 1-nm resolution in the region of 400–900 nm.

### 2.3. Determination of leaf Chl contents

After spectral measurements, 15 leaf discs of 0.5 cm^2^ from each leaf were sampled for determination of leaf Chl content. The Chl a and Chl b contents per unit area were measured spectrophotometrically using a solution of alcohol, acetone and water (4.5:4.5:1, V/V/V) as a solvent, employing the equations of Lichtenhaler and Wellburn (1983) [19]. The total Chl content was calculated as Chl*a* plus Chl*b*. The leaves that appeared evidently desiccative were not used in this study. We measured a total of 108 leaves across tillering, booting and heading stages, which included 12 leaves of the mutant and 96 leaves of the wild types.

### 2.4. Data analysis

The scatterplots of the reflectance and the first derivative (FD) reflectance *vs* Chl*a*, Chl*b* and Chl*t* were plotted, and the curves were visually analysed for extraction of spectral signatures of interest including shape, peak position, trough position and inflection point. FD was calculated with the following equation:
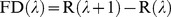
(1)where FD(λ), R(λ) and R(λ+1) represent the first derivative reflectance at wavelength λ (nm), reflectance at λ and reflectance at λ+1, respectively.

The existing published Chl-related VIs selected in this study and their formulations were summarized in Table 1 [4]–[7], [20]–[46]. Only leaf-scale indices were collected. Among the 55 indices, none were solely based on the ICCW, although 21 indices used the ICCW.

**Table 1:** The existing vegetation indices used in this study.

Index	Formulation	Reference
log(1/R737)	log(1/R737)	Yoder, Pettigrew-Crosby (1995)
SIPI	(R800-R445)/(R800-R680)	Peñuelas et al. (1995)
Ratcart	R695/R760	Carter et al. (1996)
PSSRa	R800/R680	Blackburn (1998)
PSSRb	R800/R635	Blackburn (1998)
PSNDa	(R800-R675)/(R800+R675)	Blackburn (1998)
PSNDb	(R800-R650)/(R800+R650)	Blackburn (1998)
PSSRchla	R810/R676	Blackburn (1999)
PSRI	(R680-R500)/R750	Merzlyak et al. (1999)
SR705	R750/R705	Sims, Gamon (2002)
ND705	(R750-R705)/(R750+R705)	Sims, Gamon (2002)
mND705	(R750-R445)/(R700-R445)	Sims, Gamon (2002)
mSR705	(R750-R705)/(R750+R705-2×R445)	Sims, Gamon (2002)
Readone	R415/R695	Read et al. (2002)
RGRcan	(R612+R660)/(R510+R560)	Steddom et al. (2003)
NDVIcanste	(R760-R708)/(R760+R708)	Steddom et al. (2003)
Red edge Model	(R800/R700)-1	Gitelson et al. (2005)
Green Model	(R800/R550)-1	Gitelson et al. (2005)
OSAVI	1.16×(R800-R670)/(R800+R670+0.16)	Rondeaux et al. (1996)
CI _red edge_	(R800/R700)-1	Gitelson et al. (2005)
EVI2	2.5×(R800-R660)/(1+R800+2.4×R660)	Jiang et al. (2008)
CARI	R700×(sqrt(a×670+R670+b)^2^)/R670×(a^2^+1)^0.5^ a = (R700-R550)/150 b = R550-a×550	Kim et al. (1994)
Carter^A^	R695/R420	Carter (1994)
Carter2^A^	R695/R760	Carter (1994)
Carter3^A^	R605/R760	Carter (1994)
Carter4^A^	R710/R760	Carter (1994)
Carter5^A^	R695/R670	Carter (1994)
Carter6^A^	R550	Carter (1994)
DD	(R749-R720)-(R701-R672)	Le Maire et al. (2004)
Datt^A^	(R850-R710)/(R850-R680)	Datt (1999)
Datt2^A^	R850/R710	Datt (1999)
Datt4^A^	R672/(R550×R708)	Datt (1998)
Datt5^A^	R672/R550	Datt (1998)
Datt6^A^	R860/(R550×R708)	Datt (1998)
Gitelson2^A^	(R750-R800/R695-R740)-1	Gitelson et al. (2003)
Gitelson^A^	1/R700	Gitelson et al. (1999)
mNDVI	(R800-R680)/(R800+R680-2×R445)	Sims, Gamon (2002)
Maccioni^A^	(R780-R710)/(R780-R680)	Maccioni et al. (2001)
mSR	(R800-R445)/(R680-R445)	Sims, Gamon (2002)
SRPI	R430/R680	Peñuelas et al. (1995)
NDVI2^A^	(R750-R705)/(R750+R705)	Gitelson, Merzlyak (1994)
NPCI	(R680-R430)/(R680+R430)	Penuelas et al. (1994)
REP_LE^A^	700+40×(Rre-R700)/(R740-R700) Rre = (R670+R780)/2	Cho, Skidmore (2006)
REP_Li^A^	700+40×((R670+R780/2)/(R740-R700))	Guyot, Baret (1988)
SR1^A^	R750/R700	Gitelson, Merzlyak (1997)
SR2^A^	R752/R690	Gitelson, Merzlyak (1997)
SR3^A^	R750/R550	Gitelson, Merzlyak (1997)
SR4^A^	R700/R670	McMurtey et al. (1994)
SR5^A^	R675/R700	Chappelle et al. (1992)
SR6^A^	R750/R710	Zarco-Tejada, Miller (1999)
SR7^A^	R440/R690	Lichtenthaler et al. (1996)
Sum_Dr2^A^	sum of first derivative reflectance between R680 and R780	Filella, Penuelas (1994)
Vogelmann^A^	R740/R720	Vogelman et al. (1993)
Vogelmann2^A^	(R734-R747)/(R715+R726)	Vogelman et al. (1993)
SPAD reading	Based on the transmittance at 650 nm and 940 nm	Konica Minota, Japan

The sensitivity of the VIs to Chl contents were tested with the correlation coefficients between the VIs and the Chl content, and the correlation coefficients were computed with Excel 10.0 (Microsoft).

The relationship between the VIs and the Chl content (Chl*a* or Chl*b* or Chl*t*) were fitted with linear, power, exponential, logarithmic and polynomial equations and the equation with the highest determination coefficients (R^2^) was selected as the best equation. The root mean square error (RMSE) was computed for each best equation, and the predictive performance of the VIs was assessed by ranking the RMSE values in ascending order. The relationships were fitted with Excel.

## Results

### 3.1. Rice leaf Chl content

All the leaves of both the normal N treatment and the low N treatment of the mutant ‘IG 20’ were yellow-green in color during the whole growth period. The leaves of the wild types were green in colour, although the low N treatments were shallower in leaf colour than the normal N treatments. The means and ranges of Chl content (mg/m^2^) for the 96 leaf samples of the conventional genotypes as well as Chl*a*/Chl*b* were 260.5 (148.7–378.5) for Ch*la*, 81.8 (31.9–135.3) for Chl*b*, 342.3 (209.4–497.7) for Chl*t* and 3.76 (1.99–6.55) for Chl*a*/Chl*b*. The values for the 12 leaf samples of the low-chlorophyll mutant were 52.2 (11.9–157.5) for Ch*la*, 14.7 (0.2–40.5) for Chl*b*, 66.8 (16.9–198.0) for Chl*t* and 11.08 (1.05–114.35) for Chl*a*/Chl*b*. The leaves of the wild types had an evidently higher Chl*a*, Chl*b* and Chl*t* and a much lower ratio of Chl*a* to Chl*b* than the leaves of the mutant. As compared with the previous study [6] for constructing VIs for Chl*a*, Chl*b* and Chl*t* estimation, this study had a similar mean Chl content, a lower minimum Chl content, a lower maximum Chl content, and a significantly larger variation of ratios of Chl*a* to Chl*b*.

### 3.2. Leaf spectral reflectance signatures and construction of the new VI

**Figure 1:**
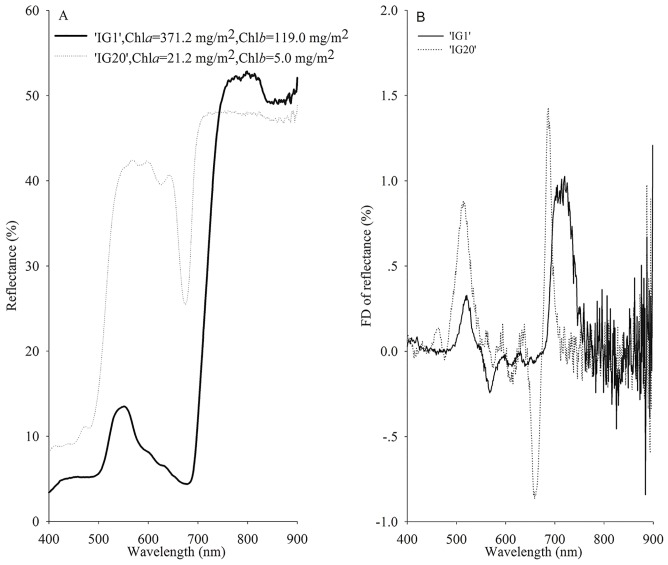
The reflectance curve (A) and the first derivative (FD) of reflectance curve (B) in the mutant (IG20) and wild type (IG1). Chl*a* and Chl*b* represent the leaf chlorophyll a content and chlorophyll b content, respectively.

As shown in Figure 1A, a profound difference in leaf spectral reflectance was observed between the conventional rice genotypes and the mutant. The reflectance curves from 640 nm to 674 nm and from 675 nm to 680 nm of the mutant leaf of a low Chl content were drastically steeper than those of the wild types in the ICCW. For both the wild types and the mutant, the inflection point of the reflectance spectra in the ICCW was 645 nm, where the FD value of reflectance started to be positive (Figure 1B). Additionally, the reflectance trough around 620 nm became evident, and the green peak around 550 nm was broadened and deformed in the reflectance spectra of the mutant as compared with that of the wild types. The reflectance spectra of all the leaves of the mutant were visually similar in shape and reflection band positions.

Based on the spectral signatures in the ICCW we observed, we found that the reflectance variation within the ICCW was sensitive to the Chl content, and constructed a new VI—the difference of first derivative sum within the ICCW (DFDS_ICCW)—for simultaneous retrieval of Chl*a*, Chl*b* and Chl*t*:
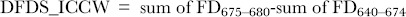
(2)


where the sum of FD_675–680_ and the sum of FD_640–674_ represent the sum of the first derivative reflectance between R675 and R680 and that between R640 and R674, respectively. R640, R674, R675 and R680 are the reflectance at 640 nm, 674 nm, 675 nm and 680 nm, respectively.

### 3.3. Sensitivity of the VIs to Chl*a*, Chl*b* and Chl*t*

Of the 55 VIs tested (Table 2), 24 were robustly sensitive to the leaf Chl*t* (r2≧0.81, n = 108), 19 were strong (0.49≦r2<0.81, n = 108), 5 were moderate (0.25≦R2<0.49) and 5 were weak (0.04≦R2<0.25). Only 2 indices, SR4^A^ and SR5^A^, were insignificantly (*P*>0.05, *n* = 108) related to the leaf Chl*a*, Chl*b* and Chl*t*. Generally, the sensitivity of the indices to Chl*t* was similar to that of Chl*a*, and the sensitivity of the indices to Chl*b* was slightly lower than Chl*t* or Chl*a*. The results showed that most of the tested indices were highly sensitive to Chl*a*, Chlb and Chl*t*.

**Table 2:** The best prediction equations of the existing vegetation indices.

Index	Prediction target	*r*	Prediction equation	R^2^	RMSE (mg/m^2^)	Rank
Log(1/R737)	Chla	0.34	y = −34230x^2^-110191x-88409	0.25	73.8	a52
	Chlb	0.40	y = −13672x^2^-43899x-35148	0.29	29.0	b47
	Chlt	0.37	y = −47901x^2^-154090x-123557	0.28	99.0	t52
SIPI	Chla	−0.65	y = 221.3x^−6.194^	0.78	59.5	a45
	Chlb	−0.51	y = 63.261x^−6.7392^	0.50	29.6	b49
	Chlt	−0.62	y = 288.46x^−6.2375^	0.76	84.1	t45
Ratcart	Chla	−0.83	y = 577.68e^−4.297x^	0.94	37.8	a11
	Chlb	−0.70	y = 2.4669x^−2.057^	0.77	15.8	b25
	Chlt	−0.82	y = 768.46e^−4.3833x^	0.94	50.9	t19
PSSRa	Chla	0.81	y = 4.3255x^1.6601^	0.90	50.4	a34
	Chlb	0.72	y = 1.7069e^0.327x^	0.72	23.2	b36
	Chlt	0.81	y = 5.2238x^1.6927^	0.89	68.1	t34
PSSRb	Chla	0.90	y = 14.01x^1.5063^	0.93	41.2	a19
	Chlb	0.90	y = 1.5702x^2^+0.682x+0.6033	0.84	13.6	b6
	Chlt	0.99	y = 16.707x^1.556^	0.95	46.9	t10
PSNDa	Chla	0.74	y = 1.3021e^6.2414x^	0.87	52.1	a37
	Chlb	0.61	y = 0.1751e^7.1639x^	0.62	26.0	b43
	Chlt	0.72	y = 1.5724e^6.335x^	0.86	72.5	t40
PSNDb	Chla	0.83	y = 9.6049e^4.182x^	0.94	38.8	a15
	Chlb	0.73	y = 717.58x^2^-555.53x+77.434	0.80	15.5	b19
	Chlt	0.83	y = 11.591e^4.2866x^	0.95	49.0	t13
PSSRchla	Chla	0.81	y = 3.9395x^1.6948^	0.90	50.4	a33
	Chlb	0.72	y = 1.6415e^0.3287x^	0.72	23.2	b37
	Chlt	0.81	y = 4.744x^1.7285^	0.89	68.0	t33
PSRI	Chla	−0.52	y = 152.13e^−13.23x^	0.61	85.8	a55
	Chlb	−0.34	y = 43.635e^−12.77x^	0.31	37.9	b55
	Chlt	−0.48	y = 198.91e^−13.064x^	0.57	120.3	t55
SR705	Chla	0.91	y = 23.775x^2.5135^	0.89	45.8	a26
	Chlb	0.88	y = 19.518x^2^-22.118x+8.0188	0.81	15.2	b9
	Chlt	0.93	y = 28.788x^2.5989^	0.91	54.2	t27
ND705	Chla	0.91	y = 572.06x^0.9776^	0.94	37.5	a7
	Chlb	0.83	y = 724.6x^2^-161.79x+13.25	0.80	15.3	b13
	Chlt	0.91	y = 758.62x^0.9945^	0.93	51.6	t21
mND705	Chla	0.90	y = 22.471x^1.5336^	0.89	47.9	a29
	Chlb	0.89	y = 0.8471x^2^+15.357x-14.561	0.80	15.5	b21
	Chlt	0.92	y = 27.138x^1.5862^	0.91	57.3	t29
mSR705	Chla	0.91	y = 494.39x^0.994^	0.94	36.8	a6
	Chlb	0.83	y = 517.31x^2^-133.21x+13.164	0.80	15.4	b15
	Chlt	0.91	y = 654.1x^1.0094^	0.93	50.7	t18
Readone	Chla	0.88	y = 1720.4x^2.5357^	0.85	54.9	a40
	Chlb	0.84	y = 838.41x^3.1792^	0.73	20.9	b34
	Chlt	0.89	y = 2403.7x^2.619^	0.87	69.2	t35
RGRcan	Chla	−0.68	y = 6638.5e^−5.523x^	0.82	68.7	a51
	Chlb	−0.53	y = 2736.3e^−6.1144x^	0.55	32.2	b54
	Chlt	−0.66	y = 8855.4e^−5.5601x^	0.79	96.6	t51
NDVIcanste	Chla	0.91	y = 609.94x^0.925^	0.94	36.6	a5
	Chlb	0.83	y = 783.43x^2^-128.31x+11.471	0.80	15.5	b16
	Chlt	0.91	y = 809.92x^0.9412^	0.93	50.3	t17
Red edge Model	Chla	0.91	y = 117.36x^0.821^	0.95	35.5	a3
	Chlb	0.88	y = 6.683x^2^+11.629x+4.7987	0.80	15.5	b17
	Chlt	0.92	y = 151.08x^0.8386^	0.95	45.6	t8
Green Model	Chla	0.91	y = 118.66x^1.0178^	0.94	37.6	a9
	Chlb	0.93	y = 4.6913x^2^+28.539x-2.6421	0.87	12.5	b2
	Chlt	0.94	y = 151.82x^1.0515^	0.96	41.2	t2
OSAVI	Chla	0.75	y = 1.556e^5.2403x^	0.88	50.2	a31
	Chlb	0.62	y = 0.2085e^6.0466x^	0.64	25.2	b40
	Chlt	0.73	y = 1.8751e^5.324 x^	0.87	69.7	t37
CI red edge	Chla	0.91	y = 117.36x^0.821^	0.95	35.5	a4
	Chlb	0.88	y = 6.683x^2^+11.629x+4.7987	0.80	15.5	b18
	Chlt	0.92	y = 151.08x^0.8386^	0.95	45.6	t9
EVI2	Chla	0.82	y = 7.4037e^1.9921x^	0.93	41.2	a20
	Chlb	0.71	y = 1.084e^2.3895x^	0.73	19.9	b32
	Chlt	0.81	y = 8.9479e^2.0371x^	0.93	53.9	t26
CARI	Chla	−0.87	y = 0.0159x^2^-5.7648x+540.52	0.79	39.2	a16
	Chlb	−0.82	y = 0.0141x^2^-3.6719x+247.15	0.80	15.2	b10
	Chlt	−0.88	y = 0.0299x^2^-9.4367x+787.67	0.83	48.0	t11
Carter^A^	Chla	−0.87	y = 1418.9e^−0.839x^	0.91	39.4	a17
	Chlb	−0.76	y = 676.39x^−3.0899^	0.74	19.2	b31
	Chlt	−0.86	y = 1941.8e^−0.86x^	0.92	49.8	t15
Carter2^A^	Chla	−0.83	y = 577.68e^−4.297x^	0.94	37.8	a12
	Chlb	−0.70	y = 2.4669x^−2.057^	0.77	15.8	b26
	Chlt	−0.82	y = 768.46e^−4.3833x^	0.94	50.9	t20
Carter3^A^	Chla	−0.85	y = 579.04e^−4.3x^	0.95	35.4	a2
	Chlb	−0.74	y = 2.4748 x^−2.051^	0.82	13.0	b4
	Chlt	−0.84	y = 774.39e^−4.4086^	0.96	43.9	t5
Carter4^A^	Chla	−0.90	y = 2561.5e^−4.845x^	0.92	38.2	a14
	Chlb	−0.81	y = 593.69x^2^-1001x+423.27	0.79	15.8	b24
	Chlt	−0.90	y = 3589.7e^−4.9847x^	0.93	45.4	t7
Carter5^A^	Chla	−0.43	y = −98.296x^2^+341.52x34.281	0.21	75.8	a53
	Chlb	−0.48	y = −57.997x+199.2	0.23	30.2	b51
	Chlt	−0.45	y = −99.659x^2^+290.02x157.36	0.22	102.6	t53
Carter6^A^	Chla	−0.88	y = 1248.3e^−0.102x^	0.91	43.6	a23
	Chlb	−0.83	y = 0.2785x^2^-18.344x+299.57	0.86	12.9	b3
	Chlt	−0.89	y = 1738.4e^−0.106x^	0.94	49.0	t12
DD	Chla	0.91	y = 171.95e^0.0753x^	0.85	41.7	a22
	Chlb	0.83	y = 0.1316x^2^+4.8546x+52.571	0.80	15.5	b20
	Chlt	0.91	y = 0.2558x^2^+15.278x+255.86	0.87	42.2	t3
Datt^A^	Chla	0.90	y = 18.526e^4.5459x^	0.90	44.5	a25
	Chlb	0.83	y = 443.78x^2^-139.88x+14.677	0.81	15.0	b8
	Chlt	0.91	y = 22.272e^4.6979x^	0.92	51.9	t23
Datt2^A^	Chla	0.89	y = 29.472x^2.8339^	0.83	57.3	a42
	Chlb	0.90	y = 17.484x^2^+14.947x-30.522	0.81	14.9	b7
	Chlt	0.92	y = 35.395x^2.9529^	0.86	69.4	t36
Datt4^A^	Chla	0.69	y = −237156x^2^+25959x-55.707	0.48	61.4	a46
	Chlb	0.81	y = 66027x^2^+7841x-38.216	0.65	20.3	b33
	Chlt	0.75	y = −171128x^2^+33800x-93.923	0.56	77.3	t42
Datt5^A^	Chla	−0.27	y = −5518x^2^+3806.5x-375.93	0.44	63.9	a49
	Chlb	−0.09	y = −2482x^2^+1820.7x-232.67	0.46	25.3	b41
	Chlt	−0.23	y = −8000x^2^+5627.2x-608.59	0.46	85.3	t46
Datt6^A^	Chla	0.86	y = 2546.3x^1.2194^	0.85	54.5	a39
	Chlb	0.92	y = −563.98x^2^+748.31x-18.395	0.86	13.0	b5
	Chlt	0.91	y = 3709.6x^1.2735^	0.89	64.0	t32
Gitelson2^A^	Chla	−0.75	y = 5.2141e^−1.03x^	0.63	83.9	a54
	Chlb	−0.74	y = 0.4714e^−1.3512x^	0.59	31.4	b53
	Chlt	−0.77	y = 5.7811e^−1.0753x^	0.66	109.9	t54
Gitelson^A^	Chla	0.88	y = 50890x^2.0381^	0.89	50.3	a32
	Chlb	0.87	y = 15333x^2^-5.1781x-4.1117	0.78	16.2	b28
	Chlt	0.90	y = 80087x^2.1079^	0.91	60.4	t31
mNDVI	Chla	0.71	y = 1.1004e^5.2579x^	0.84	56.5	a41
	Chlb	0.56	y = 0.1657e^5.8958x^	0.58	28.5	b45
	Chlt	0.68	y = 1.3561 e^5.3138x^	0.82	80.0	t43
Maccioni^A^	Chla	0.90	y = 468.03x^1.1116^	0.93	37.6	a10
	Chlb	0.81	y = 524.36x^2^-195.48x+19.343	0.79	15.8	b23
	Chlt	0.90	y = 22.432e^4.6798x^	0.93	44.9	t6
mSR	Chla	−0.32	y = −0.0202x^2^-3.6039x+105.02	0.47	62.1	a48
	Chlb	−0.14	y = −0.0073x^2^-1.1513x+37.45	0.31	28.5	b46
	Chlt	−0.28	y = −0.0275x^2^-4.7551x+142.47	0.44	87.0	t47
NDVI2^A^	Chla	0.91	y = 572.06x^0.9776^	0.94	37.5	a8
	Chlb	0.83	y = 724.6x^2^-161.79x+13.25	0.80	15.3	b14
	Chlt	0.91	y = 758.62x^0.9945^	0.93	51.6	t22
NPCI	Chla	−0.76	y = 185.21e^−5.54x^	0.90	51.3	a35
	Chlb	−0.63	y = 51.691e^−6.487x^	0.67	25.0	b39
	Chlt	−0.75	y = 240.87e^−5.6355x^	0.89	70.6	t39
REP_LE^A^	Chla	0.73	y = 2E-06e^0.0261x^	0.85	47.0	a27
	Chlb	0.59	y = 8E-08e^0.0288x^	0.56	25.5	b42
	Chlt	0.71	y = 2E-06e^0.0263x^	0.83	74.6	t41
REP_Li^A^	Chla	−0.62	y = 7E+18x^−5.741^	0.76	57.8	a43
	Chlb	−0.49	y = 3E+19x^−6.1545^	0.48	30.0	b50
	Chlt	−0.60	y = 1E+19x^−5.7714^	0.74	95.2	t50
SR1^A^	Chla	0.91	y = 20.424x^2.0298^	0.91	44.5	a24
	Chlb	0.88	y = 7.965x^2^-5.2604x+2.1228	0.80	15.3	b11
	Chlt	0.92	y = 24.674x^2.0062^	0.92	52.5	t25
SR2^A^	Chla	0.88	y = 10.593x^1.5411^	0.94	41.6	a21
	Chlb	0.83	y = 1.7565x^2^-4.8351x+9.0096	0.76	17.0	b29
	Chlt	0.89	y = 12.789 x^1.5807^	0.94	52.0	t24
SR3^A^	Chla	0.91	y = 21.529x^2.2107^	0.85	51.5	a36
	Chlb	0.93	y = 5.8111x^2^+17.204x-25.878	0.88	12.2	b1
	Chlt	0.94	y = −15.607x^2^+238.91x-238.84	0.89	38.6	t1
SR4^A^	Chla	−0.06	y = −114.37x^2^+719.91x-871.51	0.38	67.0	a50
	Chlb	−0.17	y = −37.435x^2^+226.79x-260.71	0.28	29.3	b48
	Chlt	−0.10	y = −151.81x^2^+946.7x-1132.2	0.37	92.5	t48
SR5^A^	Chla	−0.09	y = −9120.2x^2^+6433.1x-859.07	0.48	61.7	a47
	Chlb	0.05	y = −3456.2x^2^+2521.7x-367.62	0.41	26.4	b44
	Chlt	−0.05	Y = −12576x^2^+8954.8x-1226.7	0.48	83.9	t44
SR6^A^	Chla	0.92	y = 26.484x^3.1156^	0.87	47.7	a28
	Chlb	0.88	y = 41.718x^2^-58.098x+22.191	0.80	15.3	b12
	Chlt	0.93	y = 32.113x^3.2249^	0.90	57.0	t28
SR7^A^	Chla	0.90	y = 429.79x^2.2355^	0.93	39.6	a18
	Chlb	0.83	y = 288.79x^2^-165.83x+30.259	0.75	17.1	b30
	Chlt	0.90	y = 570.74x^2.2932^	0.94	49.3	t14
SRPI	Chla	0.78	y = 4.2726e^3.6528^	0.90	52.4	a38
	Chlb	0.67	y = 0.577e^4.3543x^	0.70	23.9	b38
	Chlt	0.77	y = 5.141e^3.7278x^	0.90	70.4	t38
Sum_Dr2^A^	Chla	0.75	y = 1E-05x^4.4928^	0.80	59.1	a44
	Chlb	0.59	y = 0.1296e^0.143x^	0.53	30.4	b52
	Chlt	0.72	y = 1E-05x^4.532^	0.78	94.5	t49
Vogelmann^A^	Chla	0.92	y = 29.72x^6.135^	0.86	45.6	a30
	Chlb	0.87	y = 313.02x^2^-580.26x+274.16	0.79	15.9	b27
	Chlt	0.93	y = 36.19x^6.3497^	0.88	60.3	t30
Vogelmann2^A^	Chla	−0.91	y = −10448x^2^-3992.6x+21.527	0.84	33.8	a1
	Chlb	−0.88	y = 4359.1x^2^-660.68x+5.9525	0.79	15.6	b22
	Chlt	−0.93	y = −6088.5x^2^-4653.3x+27.479	0.87	42.5	t4
SPAD	Chla	0.90	y = 1.9176x^1.3184^	0.94	37.8	a13
	Chlb	0.82	y = 2.9234e^0.0794x^	0.76	22.2	b35
	Chlt	0.90	y = 2.2727 x^1.3451^	0.93	50.1	t16

**Figure 2:**
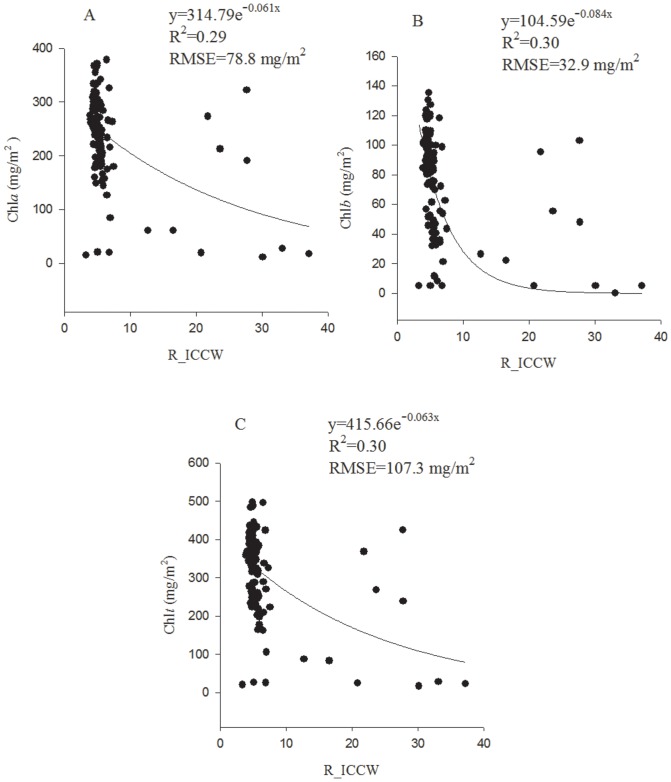
The best prediction models of R_ICCW for Chl*a* (A), Chl*b* (B) and Chl*t* (C).

**Figure 3:**
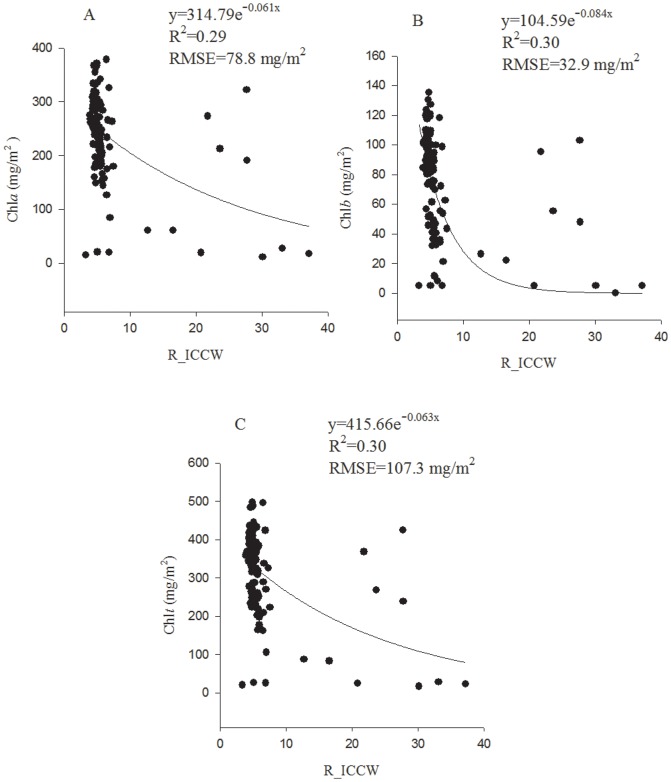
The best prediction models of DFDS_ICCW for Chl*a* (A), Chl*b* (B) and Chl*t* (C).

The mean reflectance in the ICCW (R-ICCW) was significantly (*P*<0.05) related to Chl*a*, Chl*b* and Chl*t* with a low correlation strength, yielding an *r* (*n* = 108) of −0.45, −0.40 and −0.45, respectively. In contrast, the new VI, DFDS_ICCW, had an *r* (*n* = 108) of −0.86, −0.76 and −0.85 as correlated with Chl*a*, Chl*b* and Chl*t*, respectively, indicating that this index was highly sensitive to Chl*t*, Chl*a* and Chl*b*. When leaf Chl*t* was higher than 200 mg/m^2^, the *r* value was −0.77 (*n* = 93, *P*<0.01) and −0.12 (*n* = 93, *P*>0.05) respectively between DFDS-ICCW and Chl*t* and between R_ICCW and Chl*t*. The results demonstrated that DFDS-ICCW was still highly sensitive, but R_ICCW became insensitive to Chl*t* when Chl*t* was at medium and high levels. As shown in Figure 2C, the R-ICCW tended to be saturated when leaf Chl*t*>200 mg/m^2^. Contrastingly, DFDS_ICCW decreased sensitively with the Chl*t* even when Chl*t* was higher than 200 mg/m^2^ (Figure 3C). This result confirmed the saturated reflection of the leaves at medium to high Chl content.

### 3.4. Prediction of Chl*a*, Chl*b* and Chl*t* with the best-fit equations

The best equations of R-ICCW (Figure 2) and DFDS_ICCW (Figure 3) were all exponential equations. For R-ICCW, the exponential equations yielded an RMSE (mg/g^2^) of 78.7 for Chl*a*, 32.9 for Chl*b* and 107.3 for Chl*t*. The DFDS_ICCW equations yielded an RMSE of 37.4 for Chl*a*, 16.0 for Chl*b* and 45.3 for Chl*t*. The results indicated that DFDS_ICCW had a drastically higher prediction accuracy for Chl*a,* Chl*b* and Chl*t* than R_ICCW. The prediction accuracy of DFDS_ICCW was slightly lower for Chl*b* than Chl*a* or Chl*t*.

The prediction performance with a best prediction equation for all of the 55 existing indices are presented in Table 2. Interestingly, none of the best equations were linear; they were exponential, polynomial and power. The RMSE (mg/m^2^) ranged from 33.8 to 85.8 for Chl*a*, from 12.2 to 37.9 for Chl*b* and from 38.6 to 120.3 for Chl*t*, which demonstrated that there was a large difference of prediction accuracy between the best index and the last index. However, the RMSE (mg/m^2^) of the top 30 indices ranged from 33.8 to 49.6 for Chl*a*, from 12.2 to 17.1 for Chl*b* and from 38.6 to 60.3 for Chl*t*, indicating that the differences in the RMSE were not large in the top 30 indices. An index of high predictive ability for Chl*t* (e.g. Green Model) generally also performed well for prediction of Chl*a* or Chl*b*, although the prediction accuracy for Chl*b* was generally and slightly lower than that for Chl*a* or Chl*t*, and an index of low predictive ability for Chl*t* (e.g. PSRI) was also weak for prediction of Chl*a* or Chl*b*. The SPAD reading ranked 13^th^, 35^th^ and 16^th^ among the 55 indices, respectively for prediction of Chl*a*, Chl*b* and Chl*t* with the polynomial equations, which indicated that it was also a strong index for predicting the leaf Chl contents.

**Figure 4:**
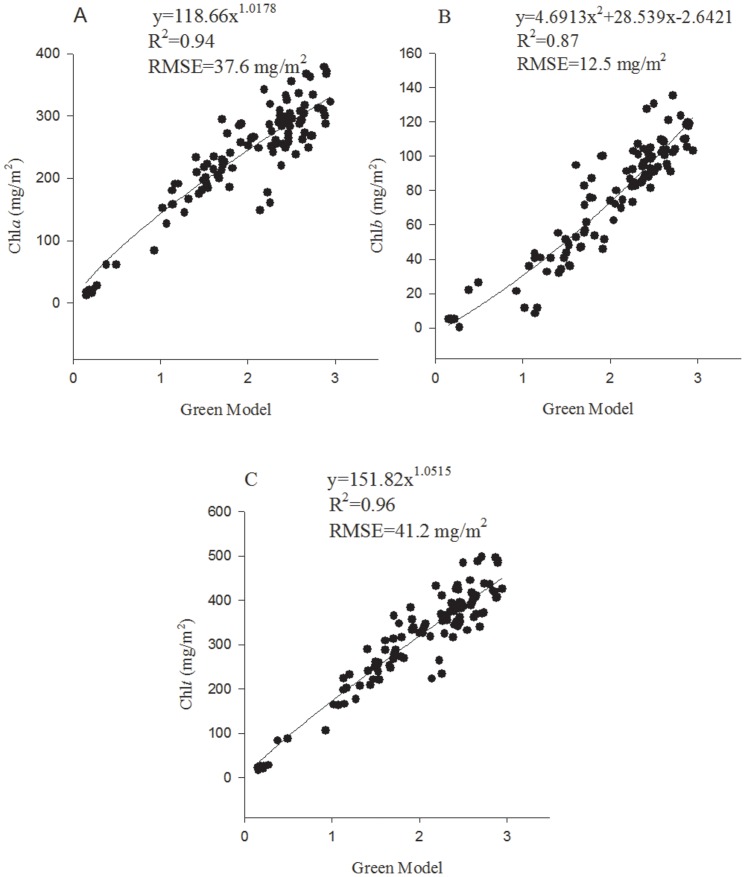
The best prediction models of Green Model for Chl*a* (A), Chl*b* (B) and Chl*t* (C).

**Figure 5:**
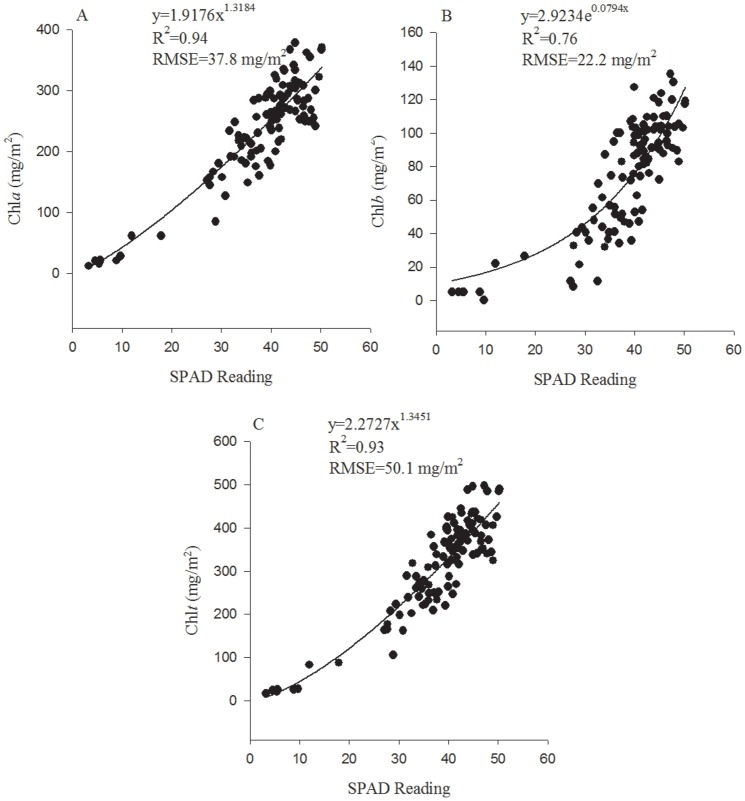
The best prediction models of SPAD readings for Chl*a* (A), Chl*b* (B) and Chl*t* (C).

The prediction results of the best VI, Green Model, together with the SPAD reading are also presented in Figure 4 and Figure 5, which confirm their high accuracy for prediction of Chl*a*, Chl*b* and Chl*t*.

The results in this study demonstrated that most of the existing indices could be used for simultaneous retrieval of Chl*a*, Chl*b* and Chl*t*.

As compared with the 55 indices, the prediction accuracy of DFDS_ICCW was similar to Datt2^A^, ranking 7^th^ for Chl*a* prediction, similar to SR6^A^ ranking 28^th^ for Chl*b* prediction and similar to Carter4^A^ ranking 7^th^ for Chl*t* prediction. The results indicated that DFDS_ICCW could simultaneously and robustly predict Chl*a*, Chl*b* and Chl*t*.

## Discussion

Most of the existing VIs as well as the SPAD reading were simultaneously and robustly or strongly related to Chl*a*, Chl*b* and Chl*t*, and achieved a high accuracy for Chl*a*, Chl*b* and Chl*t* prediction. As most of the indices were originally sought for prediction of Chl*t*, the results in this study suggested that the indices could be extended for simultaneous retrieval of Chl*a*, Chl*b* and Chl*t*. None of the best-fit equations for prediction of Chl*a*, Chl*b* and Chl*t* were linear equations; therefore, the ranking of the existing indices in this study was not in agreement with that of Main et al. (2011) [11], who used a linear equation for all indices. The VIs based on red edge (e.g. REP_LE^A^ and REP_Li^A^) ranked high for leaf Chl prediction in the previous study, but ranked low in this study. The indices excluding the ICCW generally performed better than those including the ICCW in this study, which is consistent with the previous study [11]. Particularly, both the best index for Chl*a* and Chl*t*, SR3^A^, and the best index for Chl*b*, Vogelmann2^A^, did not use ICCW. The simple ratio indices—SR4^A^ based on 670 nm in the ICCW and 700 nm and S5^A^ based on 675 nm in the ICCW and 700 nm—were the only indices that were insignificantly (*P*>0.05) related to the Chl contents. In contrast, another simple ratio index, SR3^A^ based on 550 nm and 750 nm in the OCCW, was the best index for prediction of Chl*a* and Chl*t*. In the ICCW, the reflectance curves from 640 nm to 674 nm and from 675 nm to 680 nm of the mutant leaf of a low Chl content were drastically steeper than that of the wild types of medium to high Chl content. This spectral signature could enlighten us to use the reflectance variation within the ICCW for retrieval of plant Chl content, although further studies are needed for understanding the mechanisms causing this signature. The successful detection of the reflectance variation within the ICCW in this study could be attributed to the high spectral resolution (1 nm) of the spectral photometer, as the current widely-used spectral meter, the Field Spec spectroradiometer (Analytical Spectral Devices, Boulder, CO, USA), has a spectral resolution of 3 nm in the red band.

Plant leaves tend to have saturated reflectance in the ICCW [6], [12] when leaf Chl*t* is medium to high, which has limited the use of this spectral region for non-destructive determination of leaf Chl. The results in this study also showed that the R-ICCW tended to be saturated when leaf Chl*t* was higher than 200 mg/m^2^. However, the new spectral index based on the reflectance variation within the ICCW decreased sensitively with the Chl*t* even when Chl*t* was greater than 200 mg/m^2^. The new index could rank in the top 10 for prediction of Chl*a* and Chl*t* as compared with the 55 tested indices, and also achieved a promising accuracy for Chl*b* prediction. Therefore, the results suggested that ICCW could also be used for development of robust VIs for retrieval of plant Chl contents. Unlike the existing VIs, the new index is solely based on the specific Chl adsorption band. Therefore, the retrieval of Chl by using this index may not be confounded by non-Chl factors, e.g. other pigments and leaf structure. Further studies are needed for confirmation of this finding at different scales (e.g. canopy and region) and for different plant species.

## Conclusions

Most of the 55 existing VIs could robustly or strongly and simultaneously predict Chl*a*, Chl*b* and Chl*t* in the rice leaves of a large variation of ratios of Chl*a* to Chl*b* in this study. It was found that the reflectance curves from 640 nm to 674 nm and from 675 nm to 680 nm of the mutant leaf were drastically steeper than those of the wild types in the ICCW, which implied that the reflectance variation within ICCW could be used for retrieval of Chl content. The new index based solely on the reflectance variation within the ICCW were simultaneously and strongly sensitive to Chl*a*, Chl*b* and Chl*t* and achieved a high accuracy for prediction of Chl*a*, Chl*b* and Chl*t*. The results suggested that ICCW could also be of potential for development of robust VIs for retrieval of plant Chl content with non-destructive reflectance measurement approaches.

## References

[1] Tanaka R, Tanaka A (2011) Chlorophyll cycle regulates the construction and destruction of the light-harvesting complexes. Biochimica et Biophysica Acta 1807: 968–976. 21216224 10.1016/j.bbabio.2011.01.002

[2] Porra RJ (2002) The chequered history of the development and use of simultaneous equations for the accurate determination of chlorophylls *a* and *b* . Photosynth Res 73: 149–156. 16245116 10.1023/A:1020470224740

[3] Arnon DI (1949) Copper enzymes in isolated chloroplasts. Polyphenoloxidase in *Beta vulgaris.* . Plant Physiol 24: 1–15. 16654194 10.1104/pp.24.1.1 PMC437905

[4] Chappelle EW, Kim MS, McMurtrey JE III (1992) Ratio analysis of reflectance spectra (RARS): an algorithm for the remote estimation of the concentrations of chlorophyll a, chlorophyll b and carotenoids in soybean leaves. Remote Sens Environ 39: 239–247.

[5] Blackburn GA (1998) Quantifying chlorophylls and carotenoids at leaf and canopy scales: an evaluation of some hyper-spectral approaches. Remote Sens Environ 66: 273–285.

[6] Datt B (1998) Remote sensing of chlorophyll a, chlorophyll b, chlorophyll a + b and total carotenoid content in Eucalyptus leaves. Remote Sens Environ 66: 111–121.

[7] Sims DA, Gamon JA (2002) Relationship between leaf pigment content and spectral reflectance across a wide range species, leaf structures and development stages. Remote Sens Environ 81: 337–354.

[8] Gitelson AA, Gritz Y, Merzlyak MN (2003) Relationships between leaf chlorophyll content and spectral reflectance and algorithms for nondestructive chlorophyll assessment in higher plant leaves. J Plant Physiol 160: 271–282. 12749084 10.1078/0176-1617-00887

[9] Gitelson AA, Vina A, Ciganda V, Rundquist DC, Arkebauer TJ (2005) Remote estimation of canopy chlorophyll content in crops. Geophys Res Lett 32: L08403 10.1029/2005GL022688

[10] Schlemmer M, Gitelson A, Schepers J, Ferguson R, Peng Y, et al (2013) Remote estimation of nitrogen and chlorophyll contents in maize at leaf and canopy levels. Int J Appl Earth Obs 25: 47–54.

[11] Main R, Cho MA, Mathieu R, O'Kennedy RM, Ramoelo A, et al (2011) An investigation into robust spectral indices for leaf chlorophyll estimation. ISPRS J Photogramm 66: 751–761.

[12] Thomas JR, Gausman HW (1977) Leaf reflectance versus leaf chlorophyll and carotenoids concentration for eight crops. Agron J 63: 845–847.

[13] Blackburn GA (2006) Hyperspectral remote sensing of plant pigments. J Exp Bot 58: 855–867. 16990372 10.1093/jxb/erl123

[14] Gitelson AA, Gritz Y, Merzlyak MN (2003) Relationships between leaf chlorophyll content and spectral reflectance and algorithms for non-destructive chlorophyll assessment in higher plant leaves. J Plant Physiol 160: 271–282. 12749084 10.1078/0176-1617-00887

[15] Hatfield JL, Gitelson AA, Schepers JS, Walthall CL (2008) Application of spectral remote sensing for agronomic decisions. Agron J 100: S117–S131.

[16] le Maire G, Francois C, Dufrene E (2004) Towards universal broad leaf chlorophyll indices using PROSPECT simulated database and hyperspectral reflectance measurements. Remote Sens Environ 89: 1–28.

[17] Féret JB, Francois C, Gitelson AA, Barry KM, Panigada C, et al (2011) Optimizing spectral indices and chemometric analysis of leaf chemical properties using radiative transfer modeling. Remote Sens Environ 115: 2742–2750.

[18] He QX, Zhou QF, Sun XM (2005) Strikingly high content of grain protein in solution-cultured rice. J Sci Food Agr 85: 1197–1202.

[19] Lichtenhaler HK, Wellburn AR (1983) Determination of total carotenoids and chlorophylls a and b of leaf extracts in different solvents. Biochem Soc T 11: 591–592.

[20] Yoder BJ, Pettigrew-Crosby RE (1995) Predicting nitrogen and chlorophyll content and concentrations from reflectance spectra (400–2500 nm) at leaf and canopy scales. Remote Sens Environ 53: 199–211.

[21] Peñuelas J, Baret F, Filella I (1995) Semiempirical indices to assess carotenoids chlorophyll-a ratio from leaf spectral reflectance. Photosynthetica 31: 221–230.

[22] Carter GA, Cibula WG, Miller RL (1996) Narrow-band reflectance imagery compared with thermal imagery for early detection of plant stress. J Plant Physiol 148: 515–522.

[23] Blackburn GA (1998) Quantifying chlorophylls and carotenoids at leaf and canopy scales: an evaluation of some hyper-spectral approaches. Remote Sens Environ 66: 273–285.

[24] Blackburn GA (1999) Relationships between spectral reflectance and pigment concentrations in stacks of deciduous broadleaves. Remote Sens Environ 70: 224–237.

[25] Merzlyak MN, Gitelson AA, Chivkunova OB, Rakitin VY (1999) Non-destructive optical detection of pigment changes during leaf senescence and fruit ripening. Physiol Plantarum 106: 135–141.

[26] Sims DA, Gamon JA (2002) Relationship between leaf pigment content and spectral reflectance across a wide range species, leaf structures and development stages. Remote Sens Environ 81: 337–354.

[27] Read JJ, Tarpley L, McKinion JM, Reddy KR (2002) Narrow-waveband reflectance ratios for remote estimation of nitrogen status in cotton. J Environ Qual 31: 1442–1452. 12371160 10.2134/jeq2002.1442

[28] Steddom K, Heidel G, Jones D, Rush CM (2003) Remote detection of rhizomania in sugar beets. Phytopathology 93: 720–726. 18943059 10.1094/PHYTO.2003.93.6.720

[29] Gitelson AA. Vina A, Ciganda V, Rundquist DC, Arkebauer TJ (2005) Remote estimation of canopy chlorophyll content in crops. Geophys Res Lett 32: L08403 10.1029/2005GL022688.

[30] Rondeaux G, Steven M, Baret F (1996) Optimization of soil adjusted vegetation indices. Remote Sens Environ 55: 95–107.

[31] Jiang Z, Huete AR, Didan K, Miura T (2008) Development of a two-band enhanced vegetation index without a blue band. Remote Sens Environ 112: 3833–3845.

[32] Kim MS, Daughtry CST, Chappelle EW, McMurtrey III JE, Walthall CL (1994) The use of high spectral resolution bands for estimating absorbed photosynthetically active radiation (Apar). In: Proc. Sixth Symposium on Physical Measurements and Signatures in Remote Sensing, Val D'Isere, France, January 17–21, pp. 299–306.

[33] Gitelson AA, Buschmann C, Lichtenthaler HK (1999) Thechlorophyll fluorescence ratio F735/F700 as an accurate measure of the chlorophyll content in plants. Remote Sens Environ 69: 296–302.

[34] Maccioni A, Agati G, Mazzinghi P (2001) New vegetation indices for remote measurement of chlorophylls based on leaf directional reflectance spectra. Journal of Photochemistry and Photobiology 61: 52–61. 10.1016/s1011-1344(01)00145-2 11485848

[35] Gitelson A, Merzlyak MN (1994) Quantitative estimation of chlorophyll-a using reflectance spectra: experiments with autumn chestnut and maple leaves. Journal of Photochemistry and Photobiology B: Biology 22: 247–252.

[36] Peñuelas J, Gamon JA, Fredeen AL, Merino J, Field CB (1994) Reflectance indices associated with physiological changes in nitrogen and water limited sunflower leaves. Remote Sens Environ 48: 135–146.

[37] Cho MA, Skidmore AK (2006) A new technique for extracting the red-edge position from hyperspectral data: the linear extrapolation method. Remote Sens Environ 101: 181–193.

[38] Guyot G, Baret F (1988) Utilisation de la haute résolution spectrale pour suivrel'état des couverts végétaux. In: Guyenne TD, Hunt, JJ (Eds.), Proc. Fourth International Colloquium on Spectral Signatures of Objects in Remote Sensing, ESA SP-287, Assois, France, 18–22 January, pp. 279–286.

[39] Gitelson AA, Merzlyak MN (1997) Remote estimation of chlorophyll content in higher plant leaves. Int J Remote Sens 18: 2691–2697.

[40] McMurtey III JE, Chappelle EW, Kim MS, Meisinger JJ, Corp LA (1994) Distinguish nitrogen fertilization levels in field corns (Zea maysL.) with actively induced fluorescence and passive reflectance measurements. Remote Sens Environ 47: 36–44.

[41] Zarco-Tejada PJ, Miller JR (1999) Land cover mapping at BOREAS using red-edge spectral parameters from CASI imagery. J Geophys Res 104: 27921–27933.

[42] Lichtenthaler HK, Lang M, Sowinska M, Heisel F, Miehe JA (1996) Detection of vegetation stress via a new high resolution fluorescence imaging system. J Plant Physiol 148: 599–612.

[43] Peñuelas J, Filella I, Lloret P, Munoz F, Vilajeliu M (1995) Reflectance assessment of mite effects on apple trees. Int J Remote Sens 16: 2727–2733.

[44] Filella I, Peñuelas J (1994) The red-edge position and shape as indicators of plant chlorophyll content, biomass and hydric status. Int J Remote Sens 15: 1459–1470.

[45] Vogelman JE, Rock BN, Moss DM (1993) Red-edge spectral measurements from sugar maple leaves. Int J Remote Sens 14: 1563–1575.

[46] Jin XL, Diao WY, Xiao CH, Wang FY, Chen B, et al (2013) Estimation of wheat agronomic parameters using new spectral indices. PLoS ONE 8: e72736 10.1371/journal.pone.0072736. 24023639 PMC3758323

